# Nationwide survey on the organ-specific prevalence and its interaction with sarcoidosis in Japan

**DOI:** 10.1038/s41598-018-27554-3

**Published:** 2018-06-21

**Authors:** Takeshi Hattori, Satoshi Konno, Noriharu Shijubo, Tetsuo Yamaguchi, Yukihiko Sugiyama, Sakae Honma, Naohiko Inase, Yoichi M. Ito, Masaharu Nishimura

**Affiliations:** 1 First Department of Medicine, Hokkaido University School of Medicine, Sapporo, Japan; 2 Department of Respiratory Medicine, National Hospital Organization Hokkaido Medical Center, Sapporo, Japan; 3 Department of Respiratory Medicine, JR Sapporo Hospital, Sapporo, Japan; 4 Shinjuku-Kaijo Bldg. Clinic, Tokyo, Japan; 5 Department of Pulmonary Medicine, Nerima Hikarigaoka Hospital, Tokyo, Japan; 6 Division of Respiratory Medicine, Toho University Omori Medical Center, Tokyo, Japan; 7 Department of Respiratory Medicine, Tokyo Medical and Dental University, Tokyo, Japan; 8 Department of Biostatistics, Hokkaido University Graduate School of Medicine, Sapporo, Japan

## Abstract

Previous studies attempted to characterize the subjects with sarcoidosis according to differences in sex, age, and the presence of specific organ involvement. However, significant interactions among these factors precluded a clear conclusion based on simple comparison. This study aimed to clarify the age- and sex-stratified prevalence of specific organ involvement and the heterogenous nature of sarcoidosis. Using the data of 9,965 patients who were newly registered into a database at the Ministry of Health, Labour and Welfare, Japan between 2002 and 2011, we evaluated the age- and sex-specific prevalence of the eye, lung, and skin involvement of sarcoidosis. We also attempted corresponding analysis considering multiple factors. As compared with several decades ago, the monophasic age distribution in men became biphasic, and the biphasic distribution in women, monophasic. The prevalence of pulmonary and cutaneous lesions was significantly associated with age, whereas the prevalence of ocular involvement showed a biphasic pattern. The prevalence of bilateral hilar lymphadenopathy was significantly higher, whereas the prevalence of diffuse lung shadow was significantly lower, in subjects with ocular involvement than those without ocular involvement. Corresponding analysis visually clarified the complex interactions among factors. Our results contribute to a better understanding of the heterogeneous features of sarcoidosis.

## Introduction

Sarcoidosis is a systemic granulomatous disorder of unknown etiology, and involves multiple organs. The disease occurs worldwide and affects both sexes and all ethnicities and age groups.

To better understand the heterogeneity of this disease, most previous studies have attempted to compare differences among patients, such as sex, age, and organ involvement^1–7^. However, due to the highly heterogeneous nature of the disease, the simple categorization of patients into two groups has inherent limitations, and the complex interactions among factors always need to be considered. In this regard, the detailed subtyping of patients using a large-scale survey is necessary to overcome this problem and clarify the characteristics of sarcoidosis.

In Japan, there is a digital registration system of sarcoidosis under the Ministry of Health, Labour and Welfare. Using this system, Morimoto *et al*. conducted a survey on the epidemiology of histologically proven sarcoidosis that was newly diagnosed in 2004 (n = 1,027)^8^. They showed a historical change in several clinical features of the disease over 50 years, such as an increase in female predominance and ocular involvement, and a decrease in bilateral hilar lymphadenopathy (BHL). After 2012, this system has been gradually revised and is now only used for patients with severe sarcoidosis (such as those who need high doses of systemic corticosteroids). However, before 2012, this system covered all subjects with sarcoidosis regardless of its severity. Taking advantage of this system before 2012, in the present study, we re-conducted a large-scale survey of histologically proven sarcoidosis diagnosed during 10 years from 2002 to 2011. In particular, we aimed to clarify the age- and sex-specific prevalence of the ocular, pulmonary, and cutaneous involvement of the disease. We also attempted to perform corresponding analysis considering multiple factors to better understand the complex and multidimensional nature of the disease.

## Methods

### Study subjects

The present study was performed using questionnaires for newly diagnosed sarcoidosis patients established by the public health system. In Japan, the Ministry of Health, Labour and Welfare defines an “intractable” disease as one for which studies on a national scale are necessary as the cause of the disease is unidentified; its treatment is difficult; it is a chronic disease; and its associated medical costs are high. According to this definition, sarcoidosis has been designated as an intractable disease since 1974. Attending physicians are requested to provide the clinical information using a questionnaire. The questionnaires are collected, and consent is obtained with the agreement of the expert panel in each prefecture (a total of 47 in Japan). Following the diagnosis of sarcoidosis, each prefecture and the government support the medical costs of treatment. All prefectures in Japan enter the clinical information into a central database at the Ministry of Health, Labour and Welfare. This was initially a paper-based registration system, but later moved to a digital system. Sarcoidosis was diagnosed histopathologically on the basis of the American Thoracic Society (ATS)/European Respiratory Society (ERS)/World Association of Sarcoidosis and Other Granulomatous Disorders (WASOG) statement^9^.

After 2012, this system has been gradually revised and is now only used for patients with severe sarcoidosis (such as those who need high doses of systemic corticosteroids). However, before 2011, this system covered all subjects with sarcoidosis regardless of its severity. Thus, in the present study, newly diagnosed sarcoidosis patients who were digitally registered and whose clinical information was entered into the central database at the Ministry of Health, Labour and Welfare from 2002 to 2011, were evaluated. Japan has two separate registry systems, one for newly diagnosed patients and the other for follow-up after the initial diagnosis. Thus, there should be no concerns regarding the duplicate entry of patients in our survey. Patients with granulomas of known causes and local sarcoid reactions were excluded. Those with no histopathological findings were also excluded. The enrollment into this health system had been conducted under agreement with use of the data for epidemiological survey from all subjects. The study was approved by the Clinical Research Ethics Committee of Hokkaido University Hospital (011-0188). All methods were performed with permission from the Ministry of Health, Labour and Welfare (07061)^10^, which provided the anonymous data.

### Questionnaires

Details of the questionnaire used in this study have been previously described^8^. In this study, questionnaires were analyzed, focusing on the age at diagnosis, sex, and the presence or absence of ocular, pulmonary, and cutaneous lesions. The questions on pulmonary involvement included two items: BHL and diffuse lung shadow (DLS). Information on the presence of cardiac sarcoidosis was not detailed in this questionnaire.

### Statistical analyses

Statistical analyses were performed using JMP version 13 (SAS Institute Inc., Cary, NC, USA). Categorical variables were compared using the chi-square test, and continuous variables were compared using an unpaired *t*-test. For all statistical analyses, P < 0.05 was considered statistically significant. To analyze the tendency of the prevalence of organ involvement by age, the Cochran-Armitage trend test was performed. To supplement the pre-existing knowledge on the nature and course of sarcoidosis, we also performed corresponding analysis using categorized data (age, sex, and with/without eye, BHL, DLS, and skin involvement). The patients were stratified using a cut-off age of 40 years (<40 vs. ≥40 years), based on previous reports^11,12^.

## Results

The number of sarcoidosis patients whose clinical information was digitally entered into the central database at the Ministry of Health, Labour and Welfare from 2002 to 2011 is shown in Table 1. As the system gradually switched from a paper-based system to a digital system in each prefecture, the number of patients in 2002 and 2003 was lower than that in the later years. Later, in 2004, almost all prefectures were using the digital system, but a few prefectures missed the entry to the center, which is the reason for the difference in the number of total patients in each year. Although some studies have shown a change in the clinical features of sarcoidosis in the last 50 years, the clinical features seen were quite similar within this 10-year period (Table 1).

**Table 1:** Clinical characteristics of subjects with sarcoidosis whose clinical information was digitally entered into the central database at the Ministry of Health, Labour and Welfare, which covers all prefectures in Japan, from 2002 to 2011.

Year	2002	2003	2004	2005	2006	2007	2008	2009	2010	2011
n	372	510	889	1098	916	915	1235	1480	1409	1141
Male/Female	150/222	201/309	309/580	407/691	327/589	340/575	458/777	521/959	524/885	395/746
Age (median, IQR)	47.5, 30–59	52, 33–63	52, 35–63	54, 35–63	53.5, 37–64	54, 36–65	54, 37–65	56, 39–65	56, 39–67	57, 41–66
Age (≥40 years), n (%)	226 (60.8)	338 (66.3)	596 (67.0)	758 (69.0)	656 (71.6)	629 (68.7)	887 (71.8)	1092 (73.8)	1048 (74.4)	880 (77.1)
Eye, n (%)	175 (47.0)	272 (53.3)	466 (52.4)	547 (49.8)	470 (51.3)	484 (52.9)	616 (49.9)	768 (51.9)	684 (47.1)	562 (49.3)
Skin, n (%)	94 (25.3)	166 (32.6)	301 (33.9)	367 (33.4)	289 (31.6)	289 (31.6)	405 (32.8)	469 (31.7)	454 (32.2)	356 (31.2)
BHL and/or DLS, n (%)	352 (94.6)	436 (85.5)	765 (86.1)	928 (84.5)	781 (85.3)	786 (85.9)	1073 (86.9)	1317 (89.0)	1226 (87.0)	995 (87.2)
BHL, n (%)	346 (93.0)	396 (77.7)	678 (76.3)	835 (76.1)	687 (75.0)	711 (77.7)	968 (78.4)	1191 (80.5)	1106 (78.5)	916 (80.3)
DLS, n (%)	185 (49.7)	226 (44.3)	387 (43.5)	486 (44.3)	397 (43.3)	411 (44.9)	570 (46.2)	681 (46.0)	635 (45.1)	545 (47.8)

IQR: interquartile range.

BHL: bilateral hilar lymphadenopathy, DLS: diffuse lung shadow.

**Figure 1:**
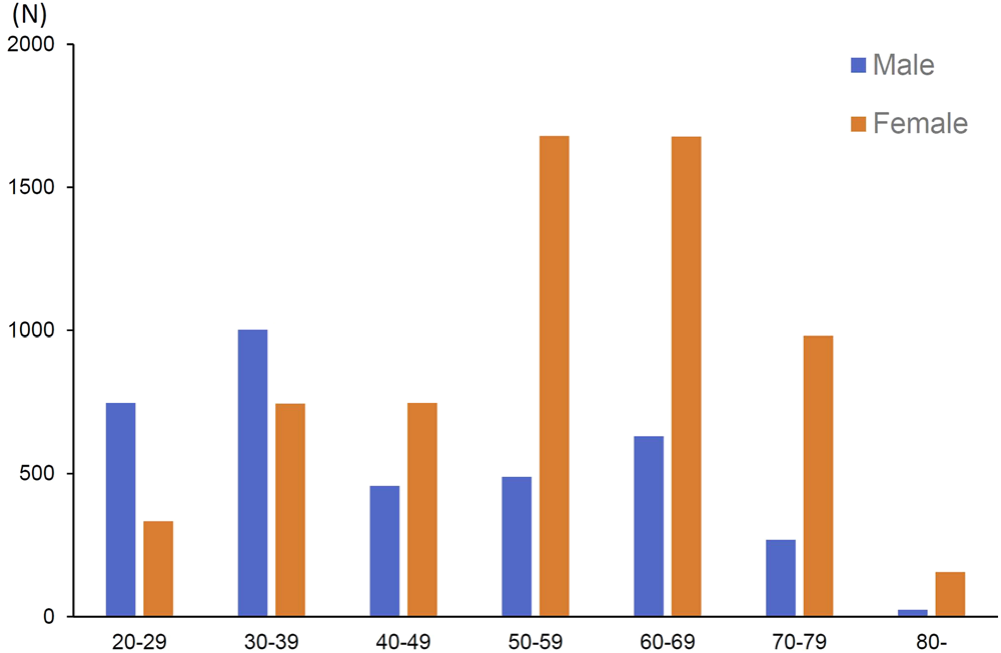
Age distribution pattern of sarcoidosis patients whose clinical information was digitally entered into the central database at the Ministry of Health, Labour and Welfare, which covers all prefectures in Japan, from 2002 to 2011.

The baseline characteristics of all the patients (n = 9,965) in this study are shown in Table 2. The patient group consisted of 3,632 men (36.5%) and 6,333 women (63.5%), with a median age at diagnosis of 54 years old. The age distribution in men revealed a biphasic pattern peaking at 30–39 and 60–69 years, whereas one peak, around 30–39 years, was shown in females (50–69 years) (Fig. 1). The overall prevalence of ocular, pulmonary (BHL and/or DLS), and cutaneous involvement was 50.4%, 86.9%, and 32.0%, respectively. Pulmonary involvement was further classified into the presence of BHL (with/without DLS) and DLS (with/without BHL), with prevalence of 78.6% and 45.4%, respectively.

**Table 2:** Baseline characteristics of subjects for analysis, whose clinical information was digitally entered into the central database at the Ministry of Health, Labour and Welfare from 2002 to 2011.

	Subjects (n = 9965)
Male/Female	3632/6333
Age (median, IQR)	54, 37–65
Age (≥40 years), n (%)	7110 (71.4)
Eye, n (%)	5024 (50.4)
Skin, n (%)	3190 (32.0)
BHL and/or DLS, n (%)	8659 (86.9)
BHL, n (%)	7834 (78.6)
DLS, n (%)	4523 (45.4)

IQR: interquartile range.

BHL: bilateral hilar lymphadenopathy, DLS: diffuse lung shadow.

**Figure 2:**
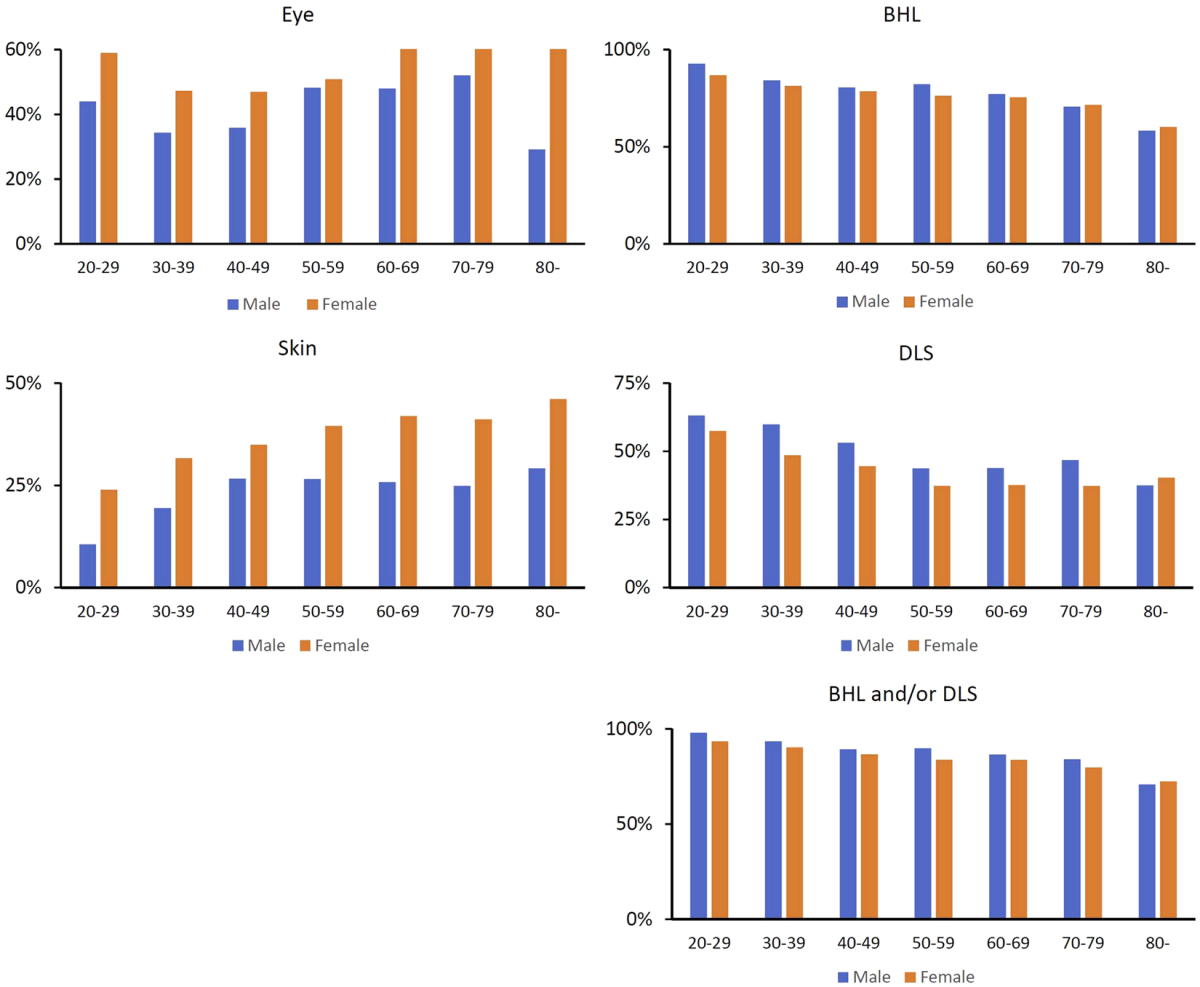
Age- and sex-specific prevalence of eye, pulmonary (BHL and/or DLS), BHL, DLS, and skin lesions. BHL; bilateral hilar lymphadenopathy. DLS; diffuse lung shadow.

We evaluated the age- and sex-specific prevalence of eye, pulmonary (BHL and/or DLS), BHL, DLS, and cutaneous lesions (Table S1). Figure 2 represents the results of Table S1 for excluded patients who were diagnosed with sarcoidosis under the age of 20 years, as only 27 patients were diagnosed in the study period. The prevalence of cutaneous lesions significantly increased with age (P < 0.001). In contrast, the prevalence of pulmonary involvement significantly decreased with age (P < 0.001). The prevalence of ocular involvement showed a biphasic pattern in both sexes, peaking at 20–29 and at 60–80 years in females, and at 20–29 and at 50–70 years in males. The prevalence of eye involvement is very high, but this is consistent with recent study^8^ which is one of the unique clinical features of sarcoidosis in Japanese patients.

**Figure 3:**
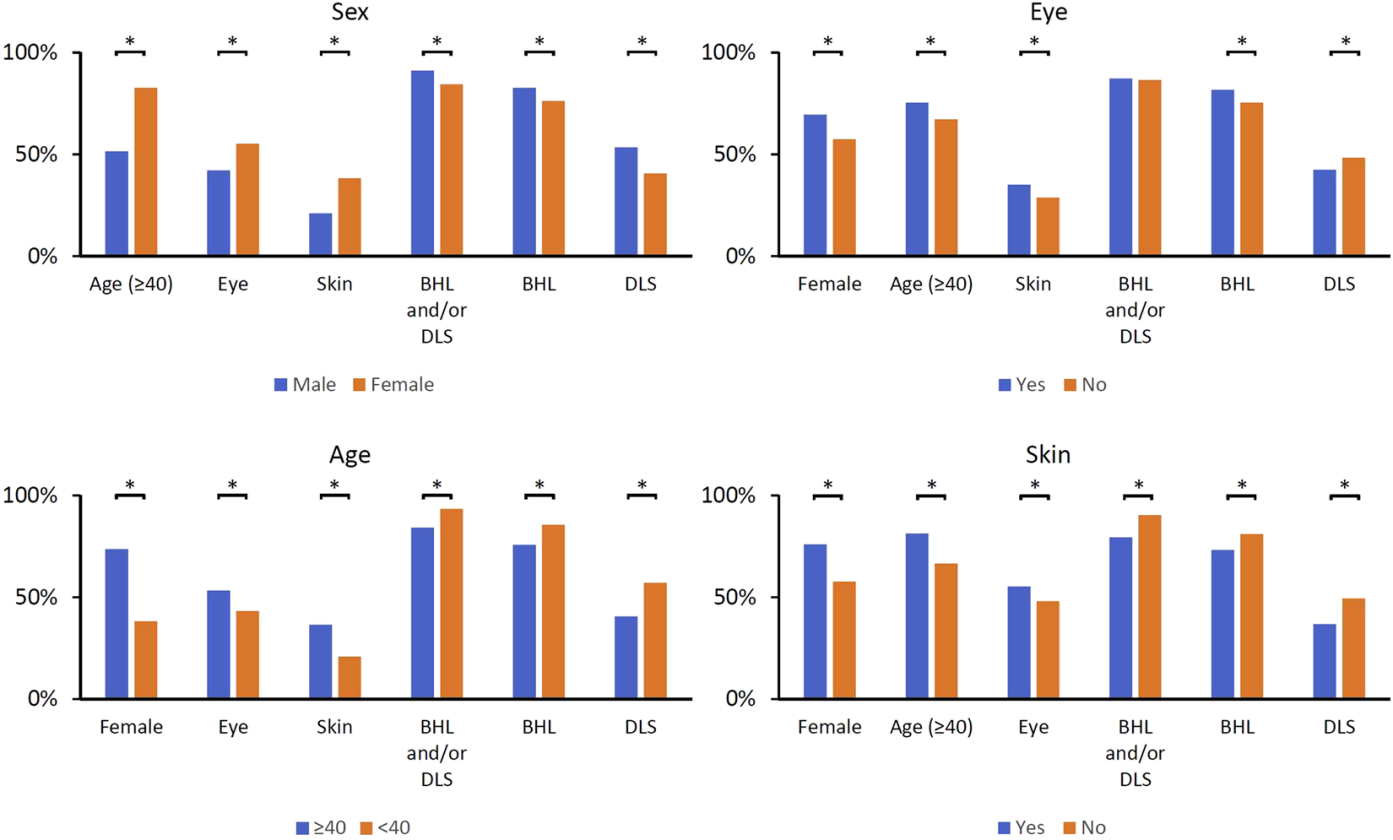
Comparison of the clinical characteristics of the patients between two groups categorized by age, sex, and the involvement of each organ [eye, pulmonary (BHL and/or DLS), BHL, DLS, and skin lesions]. BHL; bilateral hilar lymphadenopathy. DLS; diffuse lung shadow. *P < 0.001.

We next compared the clinical characteristics of the patients between two groups categorized by age, sex, and the involvement of each organ (Fig. 3 and Table S2). The elderly patients were predominantly female and had a high prevalence of ocular and cutaneous involvement and a low prevalence of lung involvement (P < 0.001). DLS was significantly more common in males (P < 0.001). The frequency of subjects with ocular involvement was significantly higher in females and in patients aged ≥40 years (P < 0.001). Ocular involvement was significantly more frequent in females (P < 0.001). Pulmonary involvement (BHL and/or DLS) was significantly more frequent in males and in patients aged ≥40 years (P < 0.001). Cutaneous involvement were significantly more frequent in females and in patients aged ≥40 years (P < 0.001). BHL and DLS were more frequent in patients aged <40 years (P < 0.001). The prevalence of cutaneous involvement was significantly lower in subjects with BHL and with DLS (P < 0.001).

**Figure 4:**
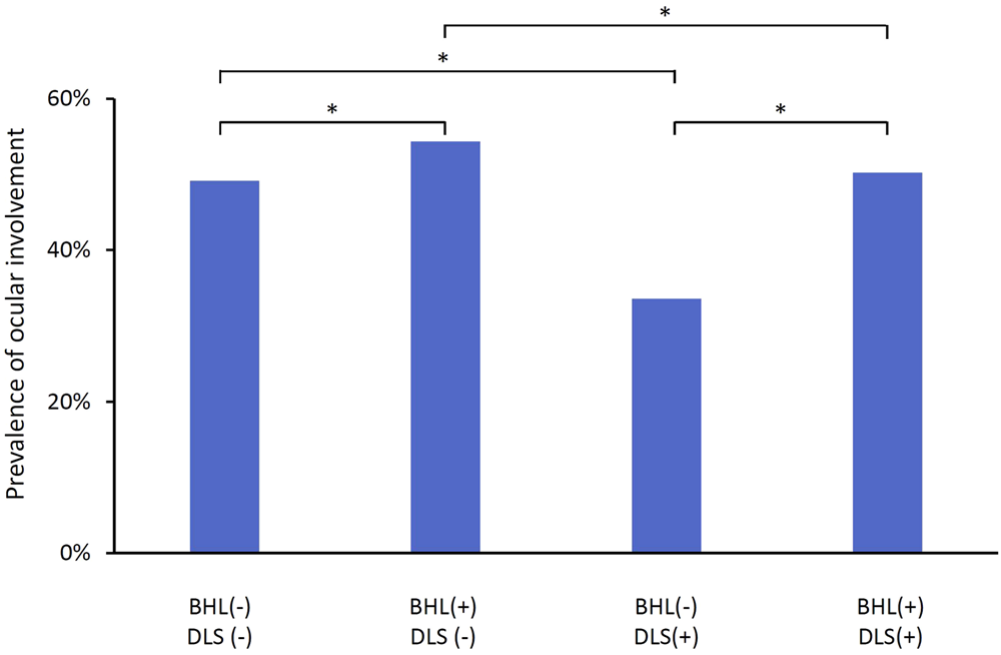
Prevalence of ocular involvement according to the presence or absence of BHL and DLS. BHL; bilateral hilar lymphadenopathy. DLS; diffuse lung shadow. *P < 0.001.

The prevalence of ocular involvement was similar with or without the pulmonary involvement (BHL and/or DLS). However, when the lung involvement was classified into the presence of BHL and DLS, the prevalence of BHL was significantly higher in subjects with ocular involvement (P < 0.001). In contrast, the prevalence of DLS was significantly lower in subjects with ocular involvement (P < 0.001). Following these contrasting associations of BHL and DLS with ocular involvement, we classified subjects into four groups according to the presence or absence of BHL and DLS, and then compared the prevalence of ocular involvement among the groups; (i) BHL (−), DLS (−) (n = 1306), (ii) BHL (+), DLS (−) (n = 4136), (iii) BHL (−), DLS (+) (n = 825), (iv) BHL (+), DLS (+) (n = 3698) The prevalence of ocular involvement was significantly higher in subjects with BHL (+), DLS (−) than in those with BHL (−), DLS (−) (Fig. 4, 54.4% vs. 49.2%, P < 0.001). In contrast, the prevalence of ocular involvement was significantly lower in subjects with BHL (−), DLS (+) than in those with BHL (−), DLS (−) (33.6% vs. 49.2%, P < 0.001).

**Figure 5:**
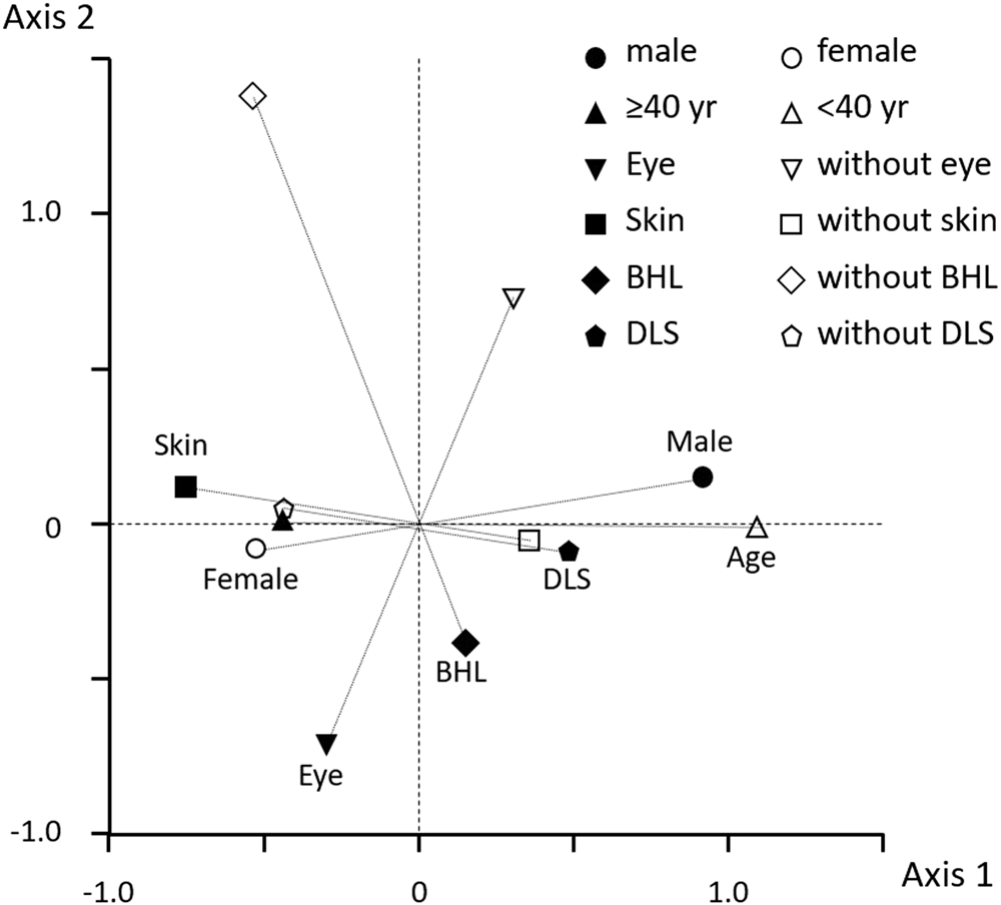
Corresponding analysis biplot of patient’s age (<40 or ≥40 years), sex, and the involvement of each organ (eye, BHL, DLS, and skin lesions). Visually, three distinct vectors were observed. Female sex, cutaneous involvement, absence of lung DLS, and the elderly patients showed the same direction of the vector. In contrast, BHL and ocular involvement showed a distinctly different direction from the other factors. BHL; bilateral hilar lymphadenopathy. DLS; diffuse lung shadow.

Figure 5 shows the results from corresponding analysis considering age, sex, and the involvement of the three organs. Visually, three distinct vectors were observed. Female sex, cutaneous involvement, absence of lung DLS, and the elderly patients showed the same direction of the vector. In contrast, BHL and ocular involvement showed a distinctly different direction from the other factors.

## Discussion

In this study, we used a nationwide registration system in Japan to conduct a large-scale survey on the age- and sex-specific prevalence of eye, pulmonary, and skin lesions of sarcoidosis. We demonstrated that ocular, pulmonary, and cutaneous involvement were highly influenced by age and sex. The prevalence of cutaneous involvement was predominant in females and significantly increased with age. In contrast, the pulmonary involvement (BHL and/or DLS) significantly reduced with age. Ocular involvement was significantly predominant in females, and showed a biphasic pattern with age. In addition, results from corresponding analysis clarified complex associations among age, sex, and the involvement of the three organs (four lesions).

Notably, the mean age in our study population (54 years old) was higher than that in several epidemiological surveys conducted several decades ago in Japan^13,14^, and this probably reflects the recent drastic aging of our population. In the previous studies, monophasic distribution in men and biphasic distribution in women had been observed in the Japanese population. However, the proportion of elderly patients has increased and, subsequently, the monophasic distribution in men has tended to become biphasic, and the biphasic distribution in women, monophasic (Fig. 1). As the clinical features of sarcoidosis are highly influenced by age, repeated epidemiological surveys are clearly needed to elucidate the longitudinal changes in any clinical manifestations of sarcoidosis, on the basis of the aging population^15,16^.

To better understand the heterogeneous features of sarcoidosis, comparisons of the clinical features of the disease (as shown in Fig. 3 and Table S2) provide helpful clinical information. Because of the aging population in Japan, a better understanding of the characteristics of elderly patients is particularly crucial. In this study, we demonstrated that elderly patients were predominantly women and had a high prevalence of cutaneous lesions and a low prevalence of pulmonary involvement, which is consistent with previous reports, which were conducted in France, including both white and black ethnicity^2,3^. Meanwhile, ocular involvement was predominant in women, which is consistent with a previous study conducted in the medical University of South Carolina^17^, but in contrast with another study conducted in the Louisiana State University^1^. This inconsistency might be because of the nature of ethnic and/or regional difference of the disease.

Since sarcoidosis is a systemic granulomatous disorder involving multiple organs, patients may be seen by the specialists in any field as well as by general physicians. Therefore, the results of our analysis on the classification of the disease based on the presence of the involvement of certain organs could provide useful information, particularly for ophthalmologists, dermatologists, and pulmonologists.

Interestingly, when the pulmonary involvement was classified into the presence of BHL or DLS, their association with ocular involvement showed opposite directions; the prevalence of BHL was higher, and in contrast, the prevalence of DLS was lower, in subjects with ocular involvement. Although the exact reason is unclear, genetic background may be involved in this interesting associations. These findings might provide us clues for better understanding of the mechanisms of development of each organ involvement of sarcoidosis.

The high heterogeneity of this disease means that a simple categorization will inherently have limitations due to the complex interactions among factors. Therefore, multivariate analysis, including corresponding analysis, which simultaneously considers all factors, can overcome this limitation and provide a better understanding of the complex associations among these factors. Visually, corresponding analysis revealed three distinct vectors (Fig. 5): female sex, cutaneous involvement, and the absence of lung DLS, and the elderly patients showed the same direction of the vector. In contrast, BHL and ocular involvement showed a distinctly different direction from the other factors. This kind of approach of multivariate analysis, such as clustering methods, has the potential to clarify the complex multidimensional nature of the disease and to identify novel phenotypes of the disease without a priori hypothesis^18–20^. Together with molecular-based measurements, including genetics and proteomics, it could be possible to identify the distinct molecular phenotypes of the disease, the so-called endotype^21^, and this should be addressed in future studies.

The strength of this study was its large-scale analysis using the digital registration system of sarcoidosis covering the whole of Japan. Analyses from large numbers of patients can overcome the heterogeneity of the disease and make it possible for a detailed classification of the disease based on sex and the 10-year age difference. There are, however, several limitations to this study. First, this study was entirely descriptive and retrospective. Second, we only evaluated the prevalence of ocular, pulmonary, and cutaneous involvement, which are relatively high prevalence in Japanese, in this study. Information on the presence of cardiac sarcoidosis was not detailed in this questionnaire and merely reported the presence of electrocardiogram (ECG) abnormalities. While we have this information, it does not reflect the presence of cardiac involvement in sarcoidosis; thus, we did not use these data in the current study. Meanwhile, the prevalence of cardiac sarcoidosis, which fulfilled the diagnostic criteria according to the Japanese Ministry of Health and Welfare guideline^22^, is relatively lower than that of other organs involved^11,12^. Thus far, we speculate that to achieve one of the goals of this study and to better understand the complex and multidimensional nature of the disease, it would be sufficient to not to include information on cardiac sarcoidosis. Third, this study focused only on histologically proven sarcoidosis, and patients who were highly suspected to have sarcoidosis but lacked pathological findings were not included. In 2002 and 2003, the number of patients was small because many prefectures had not yet switched from the paper-based system to the digital system. Thus, we cannot calculate the actual prevalence of the disease in the population at that time. However, the main goal of this study was to clarify the age- and sex-specific prevalence of the involvement of each organ and complex associations among several factors, and we believe that this limitation does not influence our findings. Although the accuracy of the diagnosis of one of the concerning issue, in Japan, all patients with sarcoidosis are diagnosed by specialists in their respective fields, including ophthalmologists, dermatologists, and pulmonologists. Lastly, due to the highly different clinical features according to ethnicity, it is not sure that the present results are applicable to other ethnic populations, thus it is worthwhile to pursue the similar approach in other areas in the world.

In conclusion, our large-scale epidemiological survey using a digital registration system revealed the age- and sex-specific prevalence of sarcoidosis and their complex interactions in the Japanese population. The findings of this study contribute to a better understanding of the heterogeneous features of sarcoidosis and the management of those with the disease^23^.

## Electronic supplementary material

Supplementary Table
