# Supplementary material for: Nationwide survey on the organ-specific prevalence and its interaction with sarcoidosis in Japan
Source: Sci Rep. 2018 Jun 21;8:9440. doi: 10.1038/s41598-018-27554-3 (PMC6013472; doi:10.1038/s41598-018-27554-3)
Supplement: Supplementary file 1 — Supplementary Table [file 41598_2018_27554_MOESM1_ESM.docx]

Supplementary information

**Nationwide survey on the organ-specific prevalence and its interaction with sarcoidosis in Japan**

Takeshi Hattori, Satoshi Konno, Noriharu Shijubo, Tetsuo Yamaguchi,

Yukihiko Sugiyama, Sakae Honma, Naohiko Inase, Yoichi M. Ito, Masaharu Nishimura

S1 Table. Age- and sex-specific prevalence of eye, pulmonary (BHL and/or DLS), BHL, DLS, and skin lesion**s**


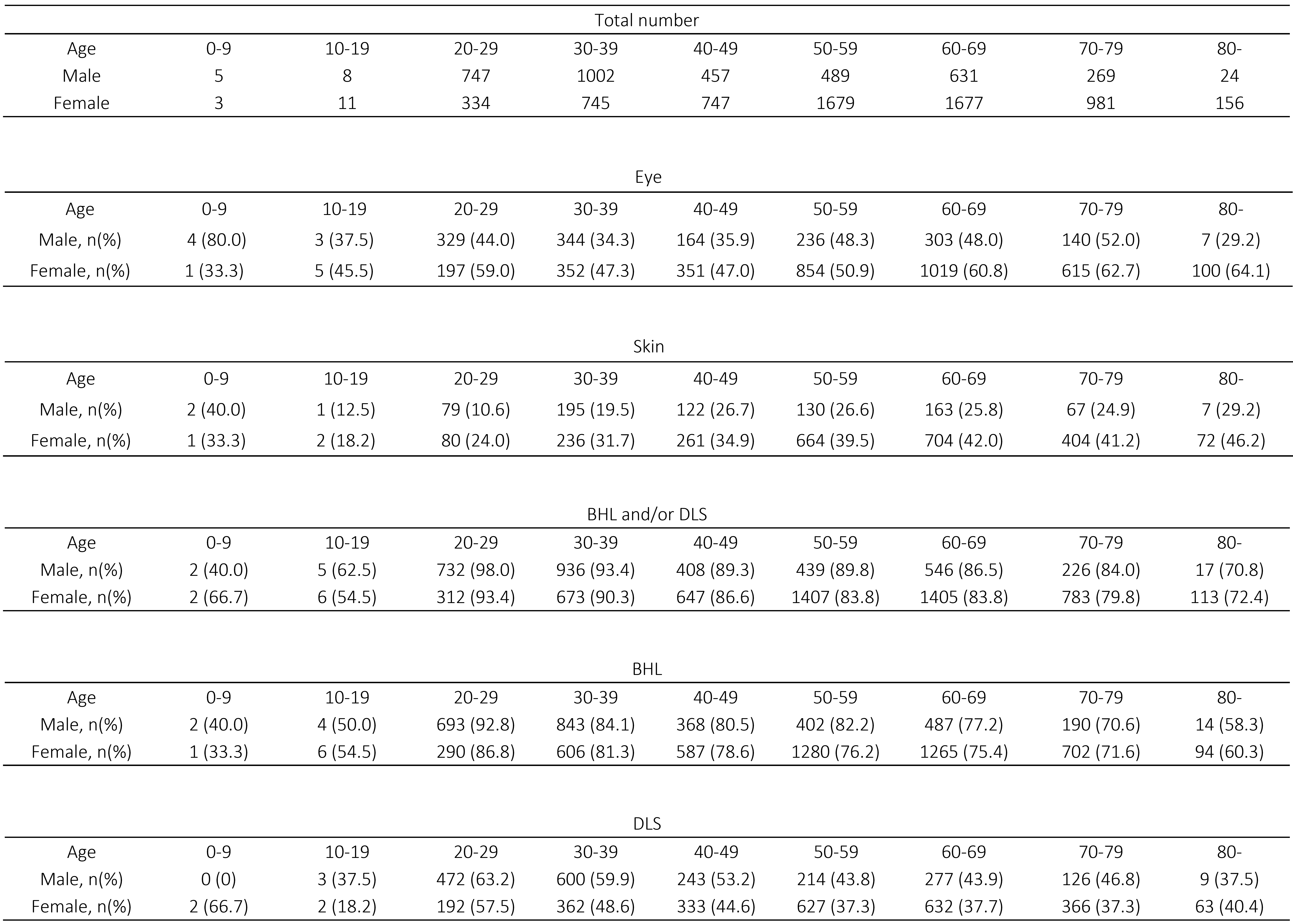


BHL: bilateral hilar lymphadenopathy, DLS: diffuse lung shadow

S2 Table. Comparison of the clinical characteristics of patients between the two groups categorized by age, sex, and the involvement of each organ


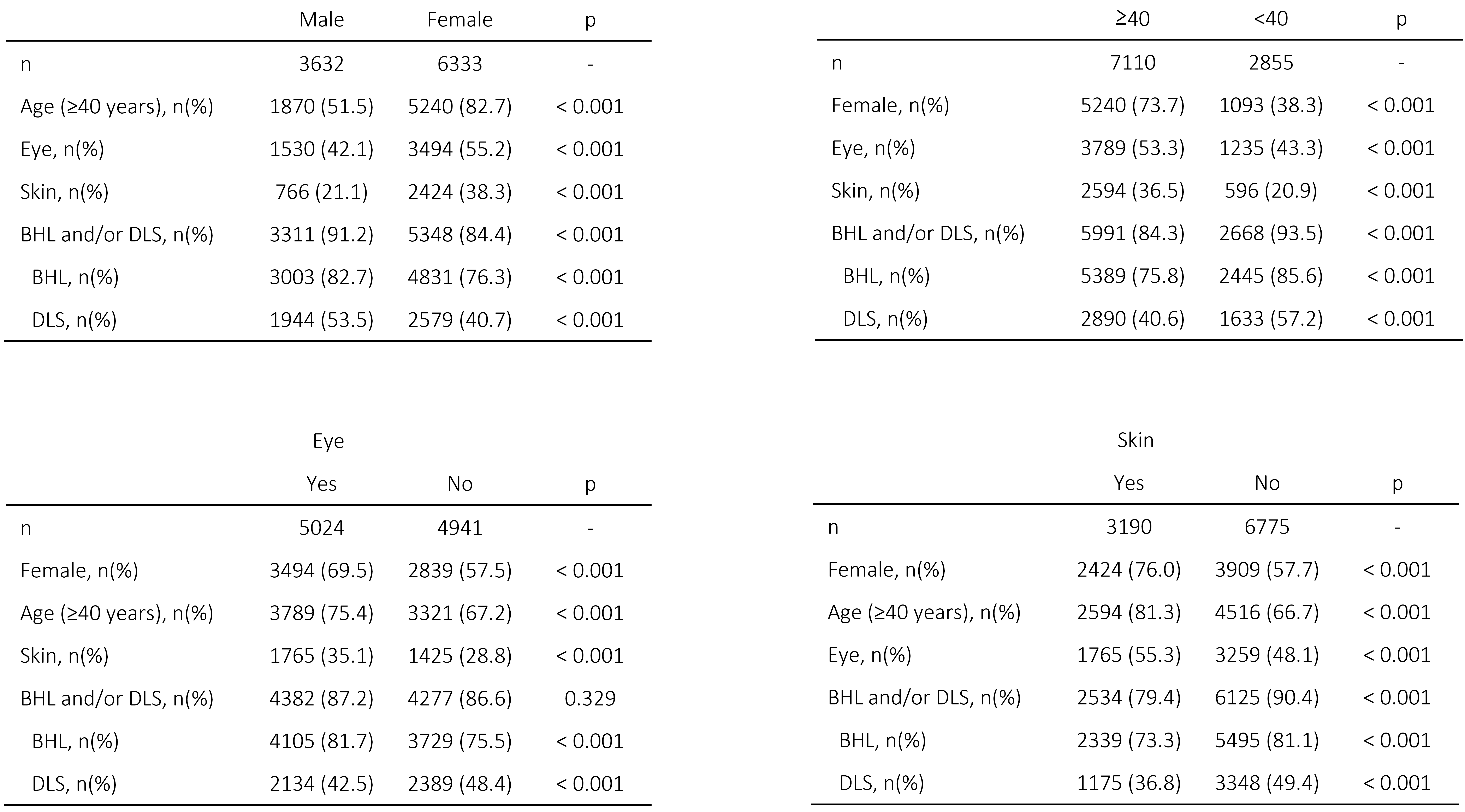


BHL: bilateral hilar lymphadenopathy, DLS: diffuse lung shadow
